# The role of LncRNAs in tumor immunotherapy

**DOI:** 10.1186/s12935-023-02872-3

**Published:** 2023-02-21

**Authors:** Xuan Pan, Chenchen Li, Jifeng Feng

**Affiliations:** Department of Medical Oncology, Jiangsu Cancer Hospital, Jiangsu Institute of Cancer Research, The Affiliated Cancer Hospital of Nanjing Medical University, Nanjing, People’s Republic of China

**Keywords:** LncRNAs, Tumor immunotherapy, PD-1, PD-L1, CTLA-4, TIM-3

## Abstract

Cancer immunotherapy is a major breakthrough in the history of tumor therapy in the last decade. Immune checkpoint inhibitors blocking CTLA-4/B7 or PD-1/PD-L1 pathways have greatly prolonged the survival of patients with different cancers. Long non-coding RNAs (lncRNAs) are abnormally expressed in tumors and play an important role in tumor immunotherapy through immune regulation and immunotherapy resistance. In this review, we summarized the mechanisms of lncRNAs in regulating gene expression and well-studied immune checkpoint pathways. The crucial regulatory function of immune-related lncRNAs in cancer immunotherapy was also described. Further understanding of the underlying mechanisms of these lncRNAs is of great importance to the development of taking lncRNAs as novel biomarkers and therapeutic targets for immunotherapy.

## Introduction

Human immune system is an important system for performing immune response and immune function, which is composed of immune organs, immune cells and immune molecules. The immune system has the function of recognizing and eliminating antigenic foreign bodies and coordinating with other systems of the body to maintain the stability of the body's internal environment and physiological balance [1]. Cancer immunotherapy, including Chimeric Antigen Receptor T-Cell Immunotherapy (CART) and immune regulatory checkpoint inhibitors, is a major breakthrough in the history of tumor therapy in the last decade [2, 3]. These are contributions of a lot of basic scientific studies in different areas including molecular biology, immunology, cell biology over the last few years, developing immune system as a therapeutic approach for cancer. Among them, cytotoxic T-lymphocyte-associated protein 4 (CTLA-4), the programmed cell death 1 (PD-1), and programmed cell death ligand 1 (PD-L1) are three important immune checkpoints, and a large number of pre-clinical basic studies and clinical trials have confirmed that antibodies designed for them can lead to tumor regression [4, 5]. Besides PD-1, PD-L1 and CTLA-4, other three T cell expressed inhibitory receptors lymphocyte-activation gene-3 (LAG-3), T cell immunoreceptor with immunoglobulin and immunoreceptor tyrosine-based inhibitory motif domain (TIGIT), T cell immunoglobulin- and mucin-domain-containing molecule 3 (TIM-3) are very promising in checkpoints immunotherapy [6].

In the past, the research related to tumor immunotherapy mainly focused on the protein-coding genes because of their biological functions in tumor immunosuppression, immune evasion, and immunotherapy resistance. As the surveillance of human transcriptomes has expanded to an unprecedented degree, our understanding of gene regulation shift substantially. LncRNAs are the most expressed non-coding RNAs in human genome, which can be located in cell cytoplasm, nucleus or exosome and interact with DNA, RNA and proteins [7, 8]. Studies have shown that lncRNAs are abnormally expressed in tumors and play an important role in tumor proliferation, angiogenesis, apoptosis and metastasis [9, 10]. In addition, a growing number of studies have suggested that lncRNAs are closely related to tumor immunotherapy through immune regulation and immunotherapy resistance [11–13]. Here, in this review, we mainly described the mechanisms of lncRNAs in regulating gene expression and well-studied immune checkpoint pathways, such as PD-1/PD-L1, CTLA-4/B7 pathway. Then, we discussed the regulatory role of lncRNAs in immunotherapy in different tumors. We hope our review will provide a theoretical basis for lncRNA as a new target for cancer immunotherapy.

## Mechanisms for the regulation of lncRNAs and mechanisms for the action of lncRNAs

With the improvement of deep RNA sequencing technology, a large number of lncRNAs have been discovered and named [14]. LncRNAs are defined as a novel class of RNAs longer than 200 nucleotides in length, covering much more loci in human genome than protein-coding genes [9]. According to the localization of lncRNAs relative to protein-coding genes on the genome, they can also be classified into five subtypes, which are long intergenic lncRNAs (lincRNAs), intronic lncRNAs, overlapping lncRNAs, sense lncRNAs, and antisense lncRNAs [15]. The biogenesis of lncRNAs includes epigenetic modification, transcription complex recruitment, RNA processing such as splicing, 5′ capping, and polyadenylation [16]. The expression and function of lncRNA is tissue-specific and cancer-specific [17]. The localization of lncRNA in cells determines its function. For example, lncRNAs in cytoplasm regulate the activity of gene transcription at the post-transcriptional level and affect downstream signaling [18]. According to the studies of the function of discovered lncRNAs, lncRNAs can regulate gene expression and protein synthesis at various levels, mainly including the following four ways.

### Epigenetic regulation

**Fig. 1:**
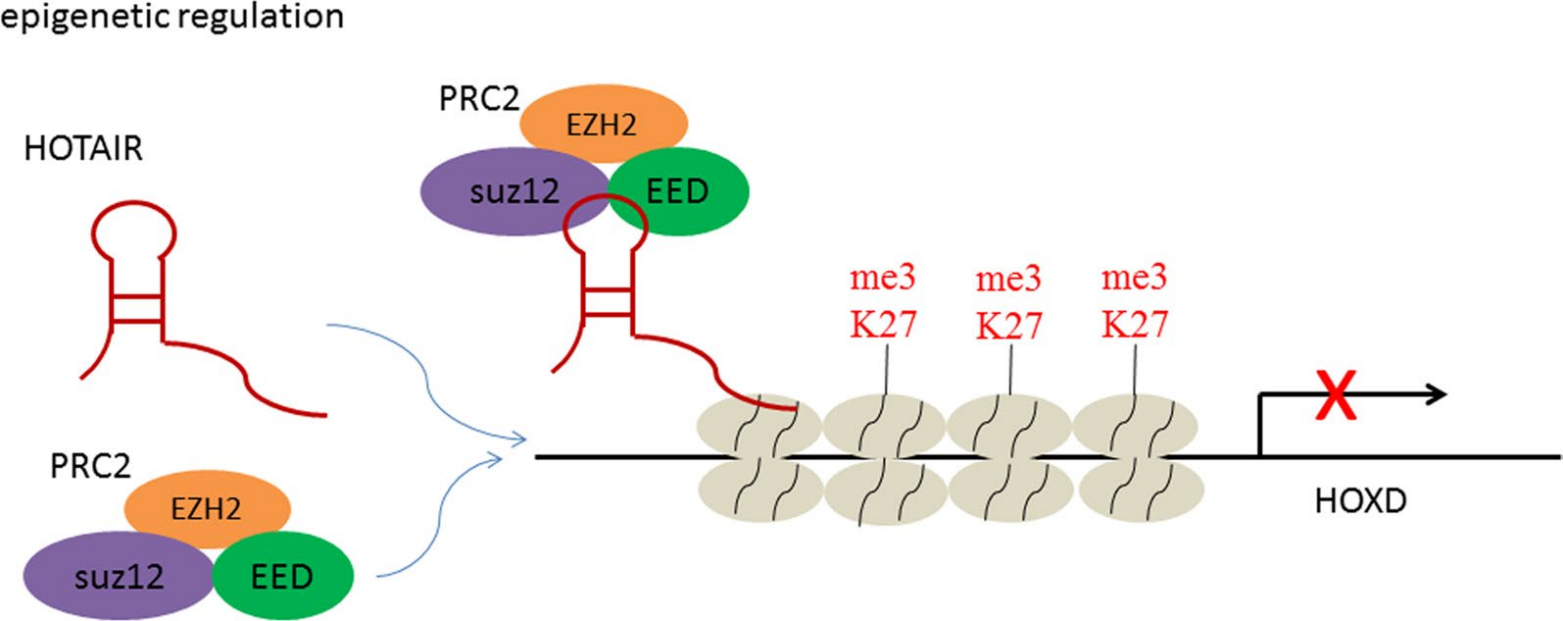
HOTAIR recruit chromatin modification complex PRC2 and induce the trimethylation of H3K27, which inhibit the expression of HOXD gene

Some specific lncRNAs can recruit chromatin reconstruction and modification complexes to specific sites, change the DNA/RNA methylation state, chromosome structure and modification state, and thus control the expression of related genes [16, 19]. A large amount of DNA/RNA methylation mutations are associated with human cancer and other diseases, and changes in chromatin modification status often affect the expression status of certain genes [20]. The most common modifications are H3K4me3, H3K9me2, and H3K27me3 in the promoter region. These histone modifications alter chromatin activity, thereby promoting or inhibiting gene transcription and controlling gene expression finally. Among these lncRNAs, the most representative one was HOX transcript antisense RNA (HOTAIR) which was transcribed by HOXC gene cluster. HOTAIR can recruit chromatin modification complex PRC2 (Fig. 1), a chromatin repressor complex that catalyzes H3K27 trimethylation, locate it to the HOXD gene cluster site, change the chromatin modification state in this region, and then inhibit the expression of HOXD gene [21]. Clinical studies have shown that the aberrant HOTAIR expression is closely related to tumor metastasis, recurrence and poor prognosis in breast cancer, colon cancer, liver cancer and other tumor tissues [22–25]. High HOTAIR level in cancer cells will inhibit the function of certain tumor metastasis suppressor genes and promote tumor deterioration, conversely, silencing HOTAIR will result in cancer cells lose their ability to metastasize [26, 27]. In addition to HOTAIR, there are other lncRNAs that can modify the epigenetic state of DNA/RNA and histones by recruiting chromatin modification complexes, such as X-inactive specific transcript (Xist) and neuroblastoma associated transcript-1 (NBAT-1) [28, 29].

### Transcriptional regulation

**Fig. 2:**
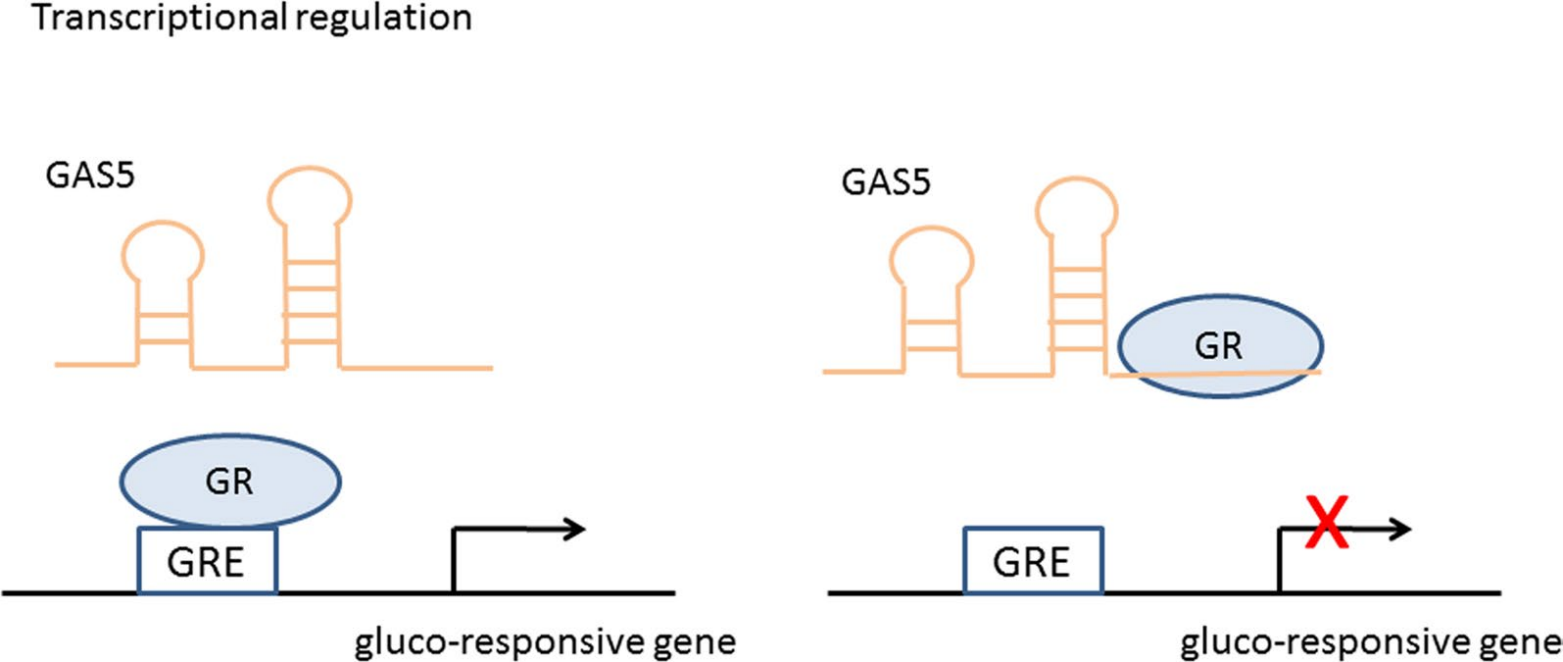
GAS5 fold into a double-stranded RNA structure and bind to the DBD of GR, thus preventing GR from binding to GRE in the glucocorticoid-responsive genes

In eukaryotic cells, transcription factors are especially important for gene transcription. They can bind to the RNA produced by gene transcription and control RNA transcription, localization, and stability. Some lncRNAs will act as ligands and combine with certain transcription factors to form complexes, then controlling gene transcriptional activity [18, 19]. For example, Metastasis-associated lung adenocarcinoma transcript 1 (MALAT1), a nuclear-retained lncRNA, plays an important role in alternative splicing of pre-mRNA. By interacting with serine/arginine splicing factors which regulate alternative splicing in a concentration- and phosphorylation-dependent manner, the MALAT1 influences the process of splicing in nuclear speckle domains [30]. Besides, a number of lncRNAs are transcription factors themselves. The glucocorticoid receptor (GR), belonging to nuclear receptor superfamily, is a hormone-dependent transcription factor [31]. Upon binding of glucocorticoid agonists, the GR is translocated from the cytoplasm to the nucleus. Through its DNA-binding domain (DBD), GR binds to glucocorticoid response elements (GREs) in the regulatory regions of glucocorticoid-responsive genes. LncRNA GAS5 could fold into a structure (double-stranded RNA GRE-mimic) and bound to the DBD of GR by acting like a decoy GRE (Fig. 2), thus preventing GR from binding to GRE in the glucocorticoid-responsive genes [32, 33]. As a result, GAS5 influenced the transcription of the glucocorticoid-responsive genes.

### Post-transcriptional regulation

**Fig. 3:**
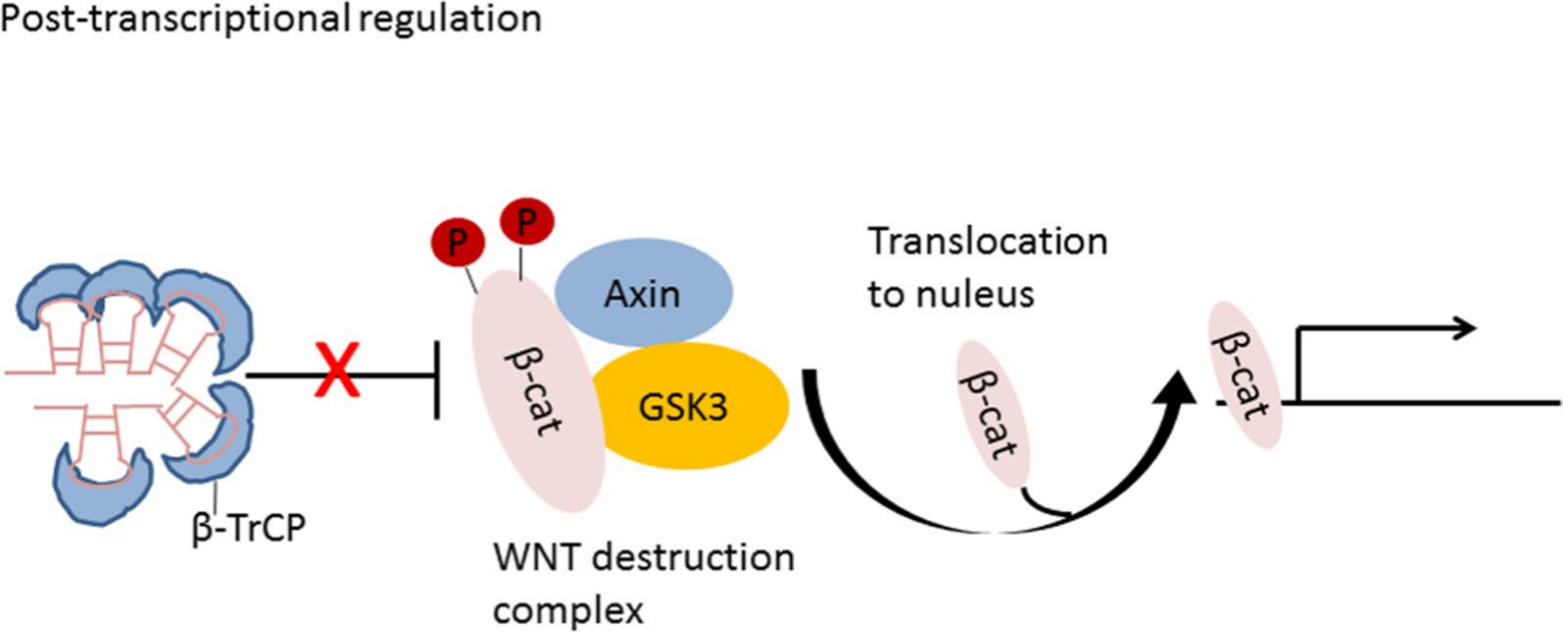
FAST forms a five stem-loop structure and binds to β-TrCP, blocking the degradation of β-catenin. Then, β-catenin translocate into the nucleus and induce activation of WNT signal pathway

In addition to the above two mechanisms, lncRNAs are also directly involved in the post-transcriptional regulation of mRNA, including variable shearing, RNA editing, protein translation and protein transport [34]. These processes are important for gene functional polymorphism. For instance, lncRNA FAST is highly expressed in human embryonic stem cells (hESCs), which is necessary for the maintenance of hESC pluripotency. FAST in cytoplasm forms a five stem-loop structure and binds to the E3 ubiquitin ligase β-TrCP, resulting in blockade of the combination between β-TrCP with phosphorylated β-catenin (Fig. 3). Then, β-catenin translocate from the cytoplasm into the nucleus and induce the transcription of WNT signal pathway-related genes required for pluripotency [35]. Antisense lncRNAs are mainly involved in post-transcriptional regulation of mRNA [36]. In the process of variable mRNA shearing regulation, antisense lncRNAs will bind to mRNA complementary regions, affect the recruitment of splines at some shearing sites, and control the mRNA shearing process. In the process of mRNA nuclear transport and intracellular localization, some antisense lncRNAs perform its regulatory function though interacting with mRNA.

### Control of microRNAs (miRNAs)

**Fig. 4:**
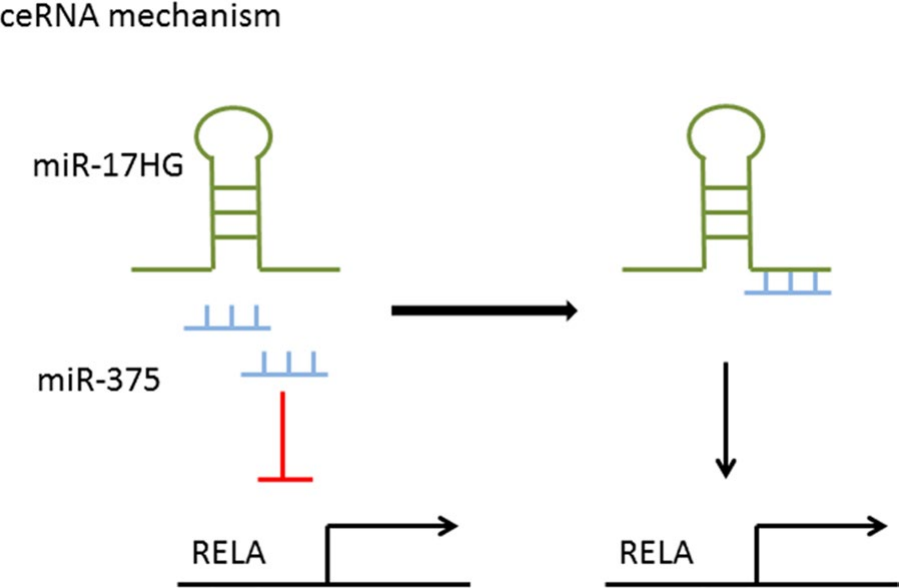
MIR17HG increases NF-κB/RELA expression by competitively sponging miR-375

In addition to directly regulating mRNA, lncRNAs can also affect the expression of their target genes by controlling miRNA expression [37]. In many tumor cells and specific tissues, lncRNAs carrying "seed sequences" of certain miRNAs bind to miRNAs like sponges, thus preventing miRNAs from binding to their target mRNAs. LncRNA MIR17HG was highly expressed in colorectal cancer. RELA is a putative downstream target of miR-375. Studies have confirmed MIR17HG increases nuclear factor kappa-B (NF-κB)/RELA expression by competitively sponging miR-375 (Fig. 4) [38].

### Immune checkpoint pathway

#### PD-1/PD-L1 signaling pathway

PD-1/PD-L1 pathway is an important immune checkpoint, and blocking the PD-1/PD-L1 pathway with anti-PD-1/PD-L1 monoclonal antibody is one of the most important advances in the history of cancer therapy [39–41]. The pathway and mechanisms involved in PD-1 and PD-L1 have been revealed by a lot of studies, which showed to be unpredictable and novel. PD-1 is a 50–55 kDa type I transmembrane glycoprotein, belonging to the immunoglobulin superfamily, which is characterized by the presence of N-terminal and C-terminal tyrosine residues in the cytoplasmic region [42]. The former is involved in the formation of an immune receptor tyrosine-based inhibitory motifs (ITIM), while the latter is involved in the formation of an immune receptor tyrosine-based switch motifs (ITSM), which plays a key role in the negative regulation of PD-1. PD-1 exists on the cell surface in the form of monomer, and was first found to be expressed in the double-negative cells of the thymus. PD-1 can also be expressed in activated T cells, B cells, natural killer cells (NK cells), dendritic cells (DCs) and activated monocytes [42, 43].

When PD-1 binds to its ligand, tyrosine phosphorylation occurs in the ITSM region of PD-1, protein tyrosine phosphatase molecules are recruited to dephosphorylate the downstream effector molecules and transduce negative signals to play a negative regulatory role. Mutation studies have shown that immunosuppressive effect of PD-1 is dependent on ITSM phosphotyrosine, which preferentially recruits phosphatase-2 in the Src homologous region 2 domain (SHP-2), leading to dephosphorylation and down-regulation of downstream signaling pathways [44–46].

What’s more, binding of PD-1 to ligand upregulates E3-ubiquitin ligases such as the Casitas B-lineage lymphoma proteins (Cbl-b and c-Cbl), triggers down-regulation of T cell receptors, and inhibits T cell activation and cytokine release [47]. The PD-1/PD-L1 interaction reduces the T cell survival through inhibiting T cell proliferation and interferon-γ (IFN-γ) production [48]. IFN-γ is a key pro-inflammatory cytokine that promotes the inflammatory activity of T cells. Reduced T cell proliferation was also associated with reduced IL-2 secretion. The overexpression of PD-1 on CD8^+^ T cells is one of the indicators of T cell exhaustion. In addition, many transcription factors trigger PD-1 expression, such as nuclear factor of activated T cells (NFAT), NOTCH, FoxO transcription factors (FoxO1), and IFN regulator 9 (IRF9) [49].

The ligands of PD-1, PD-L1 (also known as B7-H1) and PD-L2 (also known as B7-DC), are both members of the B7 family [50]. PD-L1 and PD-L2 share about 40% of the same amino acid sequence. However, the affinity of PD-L2 is 2–6 times that of PD-L1, and the expression range of PD-L1 is more extensive than that of PD-L2 [51]. PD-L1, a 33 kDa type I transmembrane glycoprotein contains two protein domains in the extracellular domain, IgV and IgC [52]. It mainly exists on the membrane of various cells in the tumor microenvironment, including T cells, B cells, DCs, macrophages, and tumor cells [53]. DCs and macrophages expressing PD-L1 may play a leading role in mediating immunosuppression of T cells. The expression of PD-L1 on these two antigen presenting cells (APC) within TME can predict therapeutic effect and outcome of PD-1/PD-L1 inhibitors [54]. PD-L1 mediated signaling was first studied in cancer cells. PD-L1 is considered to be an immune barrier that delivers anti-apoptotic signals in cancer cells, induces resistance to T-cell-mediated killing and protects cancer cells by binding to PD-1 expressed in T cells [55].

Subsequent studies have shown that PD-L1 can activate the internal signals of cancer cells independently of PD-1 and promote the proliferation and survival of cancer cells by inhibiting autophagy and mammalian target of rapamycin (mTOR) activation [56]. No significant signal transduction-related signal sequences were predicted or identified in the cytoplasmic tail of PD-L1, suggesting that PD-L1 uses a nontraditional signaling motif. Recently, three conserved sequences in the cytoplasmic tail of PD-L1 have been identified as RMLDVEKC, DTSSK, and QFEET motifs [57]. Notably, lysines 271 and 280 within the motifs of RMLDVEKC and DTSSK are thought to be targets for ubiquitination, leading to instability and down-regulation of PD-L1 [58]. The conserved RMLDVEKC motif is required to counteract the IFN-β toxicity mediated by PD-L1, while DTSSK motif acts as a negative regulator of PD-L1 function to transduce signals, and cancel IFN-related signal transduction and its cytotoxicity in cancer cells [57]. These findings mechanistically extend earlier observations that PD-L1 can act as a direct defense against cancer cells and reveal that disruption of PD-L1 expression or antibody-mediated PD-L1 blocking can make cancer cells sensitive to IFN cytotoxicity [59].

Emerging evidence has revealed the important role of lncRNAs in regulation of the immune response in tumorigenesis in part via regulation of the PD-1/PD-L1 pathway. In nasopharyngeal carcinoma (NPC), the expression of lncRNA SNHG14 was up-regulated and was positively correlated with the expression of PD-L1. The possible mechanism is that SNHG14-miR-5590-3p-ZEB1 axis positively regulates PD-L1 and promotes EMT in NPC [60]. LncRNA GATA3-AS1 predicted poor prognosis of triple-negative breast cancer (TNBC) patients. GATA3-AS1 stabilized PD-L1 protein through the deubiquitination of PD-L1, which was mechanically facilitated through miR-676-3p/COPS5 axis [61].

#### CTLA-4/B7 signaling pathway

In the early twentieth century, James Allison et al. discovered a protein called CTLA-4 and demonstrated its potent inhibitory effect in regulating T cell responses [62]. CTLA-4, also known as CD152, is a kind of leukocyte differentiation antigen and a transmembrane receptor on T cells. CTLA-4 has a close relationship with the co-stimulatory molecule receptor (CD28) on the surface of T cells in gene structure, chromosome location, sequence homology and gene expression, and can bind with the co-stimulatory molecule B7 on the surface of APCs [63]. However, CTLA-4 delivers inhibitory signals to T cells, while CD28 delivers stimulatory signals. Once CTLA-4 bound to B7 molecule as a competitive ligand, it occupies the B7 ligand binding site of APCs through its extracellular domain, resulting in CD28 signal blockade, or inhibition of T cell activation by mediating a negative signal in its intracellular domain^.^ [64]. Moreover, CTLA-4 binds B7 with greater affinity than CD28. CTLA-4 is also found constitutively expressed on regulatory T cells (Tregs), contributing to their inhibitory function. CTLA-4 on the Tregs can regulate the activity of other immune cells (APCs or naive T-cells), which is critical in the prevention of fatal autoimmunity [63]. The mice with specific deficiency of CTLA-4 in Tregs developed spontaneous systemic lymphoproliferation, severe splenomegaly, suggesting CTLA-4 exerts a crucial role in maintaining self-tolerance [65].

Initially, two humanized anti-CTLA-4 antibodies (Ipilimumab and Tremelimumab) entered clinical trials in patients with advanced cancer in 2000. Persistent tumor regression was observed in some patients with a set of toxicity due to immune checkpoint inhibitors [66, 67]. Although this phenomenon was relatively rare. Given the importance of CTLA-4 in the maintenance of homeostasis and self-tolerance, it is not surprising that anti-CTLA-4 antibodies cause a number of adverse auto-immunogenic diseases. The most common toxicities of anti-CTLA-4 antibodies include enterocolitis, inflammatory hepatitis, and dermatitis. The clinical efficacy of anti-CTLA-4 antibodies was strongest in patients with advanced metastatic melanoma, with an ORR of 15%, and continued in some patients more than 10 years after treatment ended [68].

Lots of studies have shown lncRNAs have a positive correlation with the immune checkpoint molecules such as CTLA-4, PD-1and PD-L1 in human cancers [69–71]. In hepatocellular Carcinoma (HCC), AC099850.3 was negatively correlated with NK cells, M2 macrophages, and CD8^+^ T cells in the TME. In addition, AC099850.3 was significantly positively correlated with CTLA-4 and other immune checkpoint molecules, making AC099850.3 an oncogenic role in tumorigenesis [69].

#### Other immune checkpoints

Besides PD-1/PD-L1, CTLA-4, antibodies against three promising inhibitory receptors, LAG3, TIM-3, and TIGIT expressed on tumor-infiltrating lymphocytes (TILs) are under intense clinical development [6]. LAG-3 is expressed to prevent T cell overt activation following T cells stimulation by tumor antigen [72]. Persistent antigenic stimulation within TME lead to sustained expression of LAG-3 and exhaustion of dysfunctional CD8^+^ TILs. LAG-3 and PD-1 has been observed to be co-expressed on intratumoral T cells and dual knockdown exhibit synergistical inhibition of tumor growth compared with either monotherapy [73]. TIGIT belongs to the immunoglobulin superfamily and is a member of unique family of poliovirus receptors (PVRs) including PVR (CD155), CD96, CD112 (PVRL2), CD112R (PVRIG) and CD226 (DNAM-1) [74]. Within the TME, upregulation of TIGIT is observed on CD8^+^ TILs in different cancer types. The ratio of TIGIT expression to CD226 expression on intratumoral Tregs is increased, correlated with a poor prognosis in melanoma patients [75]. TIM-3, also known as CD336, HAVCR2, is a type I transmembrane protein and constitutively expressed on a subset of Tregs. Studies demonstrated that TIM-3 cause exhaustion of T cells in cancer and chronic viral infections. Patients with upregulation of TIM-3 did not respond to treatment with cetuximab in Head and neck squamous cell carcinoma (HNSCC) [76]. Moreover, patients with upregulation of TIM-3 develop adaptive resistance to PD-1 blockade and adding TIM-3 blocking antibody after failure of PD-1 blockade has a survival advantage in NSCLC [77]. LncRNA CRNDE induced TIM-3 expression, leading to exhaustion and inhibition of anti-tumor effect of CD8^+^ T cell in oral squamous cell carcinoma (OSCC) [78]. It was reported that down-regulation of NEAT1 inhibit apoptosis of CD8^+^ T cell and enhanced the cytolysis activity of CD8^+^ T cell against HCC cells through the miR-155/TIM-3 pathway [79].

## The role of lncRNAs in immunotherapy in different tumors

New evidence suggests that various immune-related lncRNAs are involved in TME and significantly associated with immune cell infiltration and cancer cell response to anti-PD-1 immunotherapy in a variety of human cancers [11]. Based on the comprehensive analysis of lincRNA expression and tumor immune response in 9,626 tumor specimens from 32 cancer types, Weiwei Guo et al. developed a lincRNA-based immune response (LIMER) score, which can predict immune cell infiltration and the prognosis of patients in various cancer types. Their analysis also found that tumor-specific lincRNAs, including EPIC1, may modulate tumor immune responses in multiple cancer types [80]. Shouping Xu et al. identified and validated that a group of immune checkpoint-associated lncRNAs (ICP-lncRNAs) were upregulated and associated with a poor prognosis for cancer patients. These ICP-lncRNAs play a pivotal role in immune responses and immune checkpoint signaling pathways. Mechanically, HLA complex P5 (HCP5) and myocardial infarction associated transcript (MIAT), upregulated the expression of PD-L1/CD274 by sponging miR-150-5p, which is regulated by the transcriptional axis of lip-polysaccharides (LPS) -CCCTC binding factor (CTCF). MIAT knockdown combined with anti-PD-L1 antibody administration inhibited tumor growth synergistically [81]. Bone marrow-derived suppressor cells (MDSCs) participated in tumor-induced immunosuppression by significantly blocking T cell-induced antitumor responses, thereby affecting the effectiveness of tumor immunotherapy. Therapies that inhibited the differentiation and function of MDSCs could partially restore T-cell-induced antitumor immune response. The downregulation of lncRNA Pvt1 significantly altered the immunosuppressive capacity of granulocytic MDSCs (G-MDSCs) in vitro. In addition, knockdown of Pvt1 reduced the ability of G-MDSCs to accelerate tumor growth in vivo [82].

### Malignant melanoma

Malignant melanoma is one of the most aggressive cancers, proven to metastasis with a high mortality rate in the past years. With the advent of immunotherapy and targeted therapy, the prognosis of patients with malignant melanoma has improved significantly. The blockade of CTLA-4 and PD-1/PD-L1 with antibodies have been successfully used in metastatic melanoma [83]. Jianguo Zhou et al*.* identified a 15 lncRNAs signature which was an effective prognostic predictor in advanced melanoma patients administrated with anti-PD-1 antibody by weighted gene co-expression network analysis [37]. Gaopeng Li et al*.* identified a previously unknown lncRNA, capable of inducing MHC-I and immunogenicity of tumor, named LIMIT. They found that among 3,926 candidate lincRNAs annotated by GENCODE, LIMIT was enriched in hot tumor. In the human melanoma dataset, the expression of LIMIT was positively correlated with IFN-γ, MHC-I, and CD8 levels. Meanwhile, Gene Set Enrichment Analysis found that the expression of LIMIT was associated with IFN-γ responsive genes, MHC-I antigen presentation and immune activation. The expression of LIMIT was associated with survival of melanoma patients and enhancement of response rates to immune checkpoint inhibitors. They also found that LIMIT expression was associated with IFN-γ, MHC-I, and CD8 in multiple cancer types. Therefore, LIMIT is a potential immunogenic lincRNA and may be a potential target for cancer immunotherapy [12].

### Head and neck tumors

Head and neck squamous cell carcinoma (HNSCC) originates from epithelial cells in the head and neck region, including throat, pharynx, tongue, snuff and other organs. Smoking, alcohol consumption and human papilloma virus infection are important risk factors for HNSCC. Ben Ma et al. developed computational models to identify tumor-infiltrating immune-related lncRNAs (Ti-lncRNAs) in HNSCC and analyzed their relationship with clinicopathological features, molecular changes, and immunotherapy response. They demonstrated Ti-lncRNAs signature was an independent prognostic predictor in HNSCC [84]. PD-1 expression is associated with methylation in HNSCC. CpG methylation analysis in TCGA database showed that PD-1 and adjacent lncRNA AC131097.3 were co-expressed in 528 HNSCC tissues and 50 adjacent non-cancerous tissues. The expression of PD-1 mRNA and AC131097.3 were negatively correlated with promoter and positively correlated with gene body CpG methylation. AC131097.3 might play an important role in immune response in HNSCC [85]. Endogenous IFN-α-induced PD-1 and PD-L1 expression is a new immunosuppression theory in HNSCC [86]. Recently, the expression of lncMX1-215, a novel IFN-α -induced lncRNA, was found to be down-regulated in HNSCC (Table 1). LncMX1-215 expression had positive correlation with pathological grade in HNSCC. Ectopic expression of lncMX1-215 resulted in a significant inhibition of the expression of PD-L1 and galectin-9. Mechanically, lncMX1-215 directly interacts with H3K27 acetylation enzyme, GCN5, interrupt its connection with the binding sites for H3K27 acetylation on PD-L1 and galectin-9 promoters. These above results provide a new insight into immunotherapy in HNSCC [87].

**Table 1:** LncRNAs and their functions in immune-related signaling pathway

LncRNA	Type of cancer	Mechanism	Function	Immune checkpoints	References
LncMX1-215	HNSCC	Interrupting H3K27 acetylation	Inhibit proliferation and invasion	Downregulate PD-L1	[79]
SNHG14	Nasopharyngeal carcinoma	Sponge miR-5590-3p	Promote EMT	Upregulate PD-L1	[80]
IFITM4P	OSCC cytoplasm	recruit SASH1, increase NF-κB phosphorylation	Oral carcinogenesis	Upregulate PD-L1	[82]
	OSCC nucleus	reduce PTEN transcription			
CRNDE	OSCC	Sponge miR-545-5p	CD8 + T-cell exhaustion	Upregulate TIM3	[83]
PD-L1-lnc	LUAD	Enhancing c-Myc transcriptional activity	Increases proliferation and invasion		[89]
C5orf64	LUAD	NA	TME modulation	Upregulate PD-1, PD-L1 and CTLA-4	[88]
MALAT1	LUAD	Sponge miR-200a-3p	Increases proliferation and invasion	Upregulate PD-L1	[93]
CASC11	HCC	Activation of NF-κB and PI3K/AKT/mTOR pathway	Promote proliferation, mobility, and glucose metabolism	Upregulate PD-L1	[95]
SNHG3	HCC	Sponge miR-214-3p	Immune cell infiltration	Upregulate PD-L1	[97]
Ac099850.3	HCC	Target PRR11/PI3K/AKT pathway	Promote proliferation and invasion	Upregulate PD-1, PD-L1, PD-L2, and CTLA4	[98]
PCED1B-AS1	HCC	Sponge miR-194-5p	Induce PD-Ls-mediated immunosuppression	ipregulate PD-L1, PD-L2	[99]
MIAT	HCC	NA	Promote immune escape	Upregulate PD-1, PD-L1, and CTLA4	[96]
RP11-424C20.2	HCC	Sponge miR-378a-3p	Regulate immune infiltration		[100]
KCNQ1OT1	HCC	Sponge miR-506	Contribute immune escape	Upregulate PD-L1	[101]
ANRIL	HCC	Target miR-203	Decrease the percentages of NK cells and T cells		[102]
NEAT1	HCC	Sponge miR-155	Inhibit the antitumor activity of CD8 + T cell	Upregulate TIM	[103]
LINK-A	TNBC	Enhance degradation of the PLC, Rb and p53	Downregulate cancer cell antigen presentation and intrinsic tumor suppression		[107]
GATA3-AS1	TNBC	Sponge miR-676-3p	Inhibit PD-L1 ubiquitination	Upregulate PD-L1	[108]
NKILA	Breast cancer	Inhibiting NF-κB activity	Sensitize T cells to activation-induced cell death		[110]
FENDRR	Breast cancer And CRC	Enhance the inflammatory and WNT signaling pathways	Immune activation		[111]
TM4F1-AS1	Stomach adenocarcinoma	NA	Decrease proportion of CD8 + T cells		[113]
SNHG15	Stomach adenocarcinoma	Sponge miR141	Promote immune escape	Upregulate PD-L1	[114]
MIR17HG	CRC	Sponge miR-375, decrease NF-κB/RELA expression	Promote metastasis		[37]
SNHG29	CRC	Degradation of YAP protein	Promotes anti-tumor immunity	Upregulate PD-L1	[118]
LINC00657	CRC	Sponge miR1224-3p and miR-338-5p	Impair the cytotoxicity of CD8 + T cells		[119]
LINC00657	CRC	Sponge miR-203a	Promotion of stem cell-like cell invasion ability		[120]
NEAT1	GBM	Induce NF-κB signal pathway	Promoted immune escape	Upregulate PD-L1	[123]
PCAT6	CCA	Sponge miR-326	Induce the accumulation of ROS, mitochondria and metabolic dysfunction in macrophages		[124]

The expression of SNHG14 is up-regulated in nasopharyngeal carcinoma (NPC). Knockdown of SNHG14 significantly inhibited epithelial–mesenchymal transition (EMT) in NPC. The expression of Zinc finger E-box binding homeobox 1 (ZEB1) was positively correlated with that of SNHG14, and negatively correlated with that of miR-5590-3p. In addition, ZEB1 was found to upregulate PD-L1 and facilitate EMT, while SNHG14 promoted EMT of NPC by regulating PD-1 and PD-L1 in vivo. Thus, SNHG14-miR-5590-3p-ZEB1 axis positively regulates PD-L1 and promotes EMT in NPC [60]. Yanyan Tang et al. evaluated the expression of AFAP1-AS1 and PD-1 in 96 paraffin-embedded NPC specimens and confirmed that AFAP1-AS1 and PD-1 were co-expressed in infiltrating lymphocytes of NPC tissues. NPC patients with higher expression of AFAP1-AS1 or PD-1 in infiltrating lymphocytes were more prone to distant metastasis, and patients with co-expression of AFAP1-AS1 and PD-1 had the worst prognosis. These results suggest that AFAP1-AS1 and PD-1 may be potential therapeutic targets for NPC. Patients with higher AFAP1-AS1 and PD-1 expression may benefit from anti-PD-1 immunotherapy and be chosen as ideal candidates for clinical trials [88].

Oral leukoplakia (OL) is a precancerous lesion of oral squamous cell carcinoma (OSCC). LncRNA IFITM4P was highly expressed in OSCC. In cytoplasm, IFITM4P acts as a scaffold to promote SAM and SH3 domain containing1 (SASH1) recruitment and phosphorylation of TAK1 (Thr187), which induce the phosphorylation of NF-κB (Ser536) and expression of PD-L1. The series of cascade reaction lead to activation of the immunosuppressive program ultimately, allowing OL cells to escape the anticancer immunity. In the nucleus, IFITM4P decreased the transcription of PTEN by enhancing the binding of KDM5A to the PTEN promoter, thus activating PD-L1 in OL cells. Tumor-bearing mice with higher IFITM4P expression showed significantly more sensitive to anti-PD-1 immunotherapy. Collectively, IFITM4P can be used as a new therapeutic target during oral carcinogenesis [89]. TIM-3 is an immune checkpoint inhibitor and important in CD8^+^ T cell exhaustion. LncRNA CRNDE was upregulated and had a negatively correlation with IFN-γ production in tumour-infiltrating CD8^+^ T cells isolated from OSCC patients. TIM-3 was a downstream target of miR-545-5p. CRNDE induced TIM-3 through sponging miR-545-5p, thus leading to CD8^+^ T-cell exhaustion. The regulatory network plays an important role in immune escape of OSCC [78].

### Lung cancer

The development of specific antibodies against PD-1, PD-L1 and CTLA-4 has greatly prolonged the survival of patients with advanced non small cell lung cancer (NSCLC) [90]. Recently, the role of lncRNAs has been well characterized in NSCLC [91]. In our previous research, we found lncRNA KTN1-AS1 promoted the progression of NSCLC via sponging miR-130a-5p and activation of PDPK1 [92]. What’s more, lncRNAs in immune-related pathways play important roles in immune regulation in lung cancer. Yongsheng Li et al. introduced a computational algorithm, ImmLnc, to systematically identify lncRNA expression profiles. ImmLnc helps prioritizing cancer-related lncRNAs and distinguishing molecular subtypes (proliferative, intermediate and immunological) of NSCLC according to distinct immunological characterization. The classification of molecular subtypes is determined by differences in immune cell infiltration, tumor mutation burden (TMB), immunoregulatory gene expression, response to chemotherapy, and prognosis [13].

MDSCs, usually derived from bone marrow progenitor cells, are heterogeneous cell populations composed of immature granulocytes, DCs, macrophages and early undifferentiated bone marrow progenitors. LncRNA SNHG6 was more expressed in tumor-derived MDSCs than in spleen-derived MDSCs in Lewis lung cancer xenograft mice. SNHG6 promoted the differentiation of CD11b^+^Ly6G^−^Ly6C^high^ mononuclear MDSCs (Mo-MDSCs), but not CD11b^+^Ly6G^+^Ly6C^low^ polymorphonuclear MDSCs (PMN-MDSCs). However, SNHG6 did not affect the immunosuppressive function of MDSCs. It is noteworthy that SNHG6 inhibited EZH2 expression at the protein level but not at the mRNA level during MDSCs differentiation from mouse bone marrow cells. EZH2 may be a key factor in the regulation of Mo-MDSCs differentiation by SNHG6. Therefore, their findings may provide new ideas and directions for immunotherapy [93]. LncRNA C5orf64 was found to be related with TME, the abundances of tumor-infiltrating immune cells (TIICs) in lung cancer. M2 macrophages, monocytes, eosinophils and neutrophils were positively correlated with C5orf64 expression, while other two kinds of TIICs (Tregs and plasma cells) were negatively correlated with C5orf64 expression. Moreover, C5orf64 had a positive correlation with PD-1, PD-L1, CTLA-4 and a negative correlation with TP53 mutation frequency. C5orf64 could be a potential indicator for TME modulation [94].

A recent study identified that a lncRNA isoform of PD-L1 (PD-L1-lnc) was generated through alternative splicing despite PD-L1 protein was positive or negative in human lung adenocarcinoma (LUAD). The expression of PD-L1 mRNA and PD-L1-lnc was significantly upregulated by IFN-γ in LUAD cell lines [95]. PD-L1-lnc induced proliferation and invasion of LUAD cells and inhibited apoptosis of them both in vitro and in vivo through directly binding to c-Myc and enhancing its transcriptional activity. These results provide theoretical basis that PD-L1-lnc depletion in combination with PD-L1 blockade can be used as a new target in lung cancer immunotherapy [95]. In LUAD patients, the low expression of lncRNA ADAMTS9-AS2 was correlated with N stage, gender, number of smoking packs and smokers, and poor overall survival (OS). ADAMTS9-AS2 expression was associated with certain immune infiltrating cells and may be a potential biomarker of prognosis and immunotherapy response for LUAD [96]. It is widely known ubiquitin-conjugating enzyme 2C (UBE2C) plays a key role in tumor progression. Recent research confirmed the expression of UBE2C was significantly upregulated in human cancers and associated with clinicopathological characteristics, microsatellite instability (MSI), TMB, immune cell infiltration and multidrug sensitivity. UBE2C is a downstream target of miRNA-140-3p. Fundamental experiments showed lncRNA SNHG1 sponged miR-140-3p as a ceRNA to increase UBE2C expression in NSCLC cell lines. Thus, SNHG1 acts as an oncogene in NSCLC. The study elucidates regulatory mechanisms of METTL3/SNHG1/miRNA-140-3p/UBE2C axis in tumor progression and immune response in NSCLC [97].

Evidence show that LINC01140 is upregulated in various cancers. LINC01140 promotes proliferation, migration and invasion in vitro by directly interacting with miR-33a-5p and miR-33b-5p, thereby contributing the expression of c-Myc and inhibiting cisplatin-induced apoptosis. LINC01140 knockdown significantly reduced tumor growth and lung metastasis in vivo. In addition, LINC01140 directly inhibited the expression levels of miR-377-3p and miR-155-5p, leading to upregulation of their common downstream target PD-L1. Notably, researchers demonstrated that LINC01140 gene knockout and cytokine-induced killer (CIK) cells administration inhibited the growth of subcutaneous lung cancer xenografts by reducing PD-L1 expression in immunodeficient mice. In conclusion, LINC01140 protects c-Myc and PD-L1 activity from restraining antitumor miRNAs, and contributes to immune escape of lung cancer cells [98]. In NSCLC, the expression of lncRNA MALAT1 had a positive correlation with PD-L1 and a negative correlation with miR-200a-3p. MALAT1 promoted proliferation, invasion and inhibited apoptosis in NSCLC cell via sponging miR-200a-3p [99]. Thus, MALAT1 promoted progression of NSCLC by regulating miR-200a-3p/PD-L1 axis. Chronic exposure to arsenic can cause lung cancer. Arsenic induced BEAS-2B transformation was used as a model system to study the upregulation of PD-L1 by arsenic. Experimental data suggested that lncRNA LNC-DC and signal transduction and transcription activator 3 (STAT3) mediate up-regulation of PD-L1 by arsenic [100].

### Hepatocellular carcinoma (HCC)

LncRNA CASC11 was upregulated in HCC tissues and associated with poor prognosis in patients with HCC. CASC11 and E2F1 affect the activation of NF-κB signaling pathway and Phosphoinositide 3-kinase (PI3K)/AKT/mTOR pathway, thereby regulating the expression of PD-L1, which is an important target of immunotherapy [101]. TIMER database analysis indicated the expression of lncRNA MIAT in HCC had a positive correlation with the number of immune cells such as B cells, T lymphocytes, macrophages, and the expression of immune checkpoint molecules such as PD-1, PD-L1, and CTLA-4. MIAT was mainly distributed in tumor, and enriched in FOXP3 + CD4 + T cells and PDCD1 + CD8+, GZMK + CD8 + T cells through single cell sequencing analysis, indicating that it palyed an important role in immune escape of HCC [71]. Furthermore, the expression of MIAT had a correlation with the sensitivity of many anticancer drugs, especially sorafenib.

By integrating and analyzing the multiple databases, nine lncRNAs and five miRNAs were found to be significantly overexpressed in HCC tissues of recurrent patients. Among them, SNHG3, LINC00205, ASF1B, AURKB, CCNB1, CDKN3 and DTL were also closely correlated with HCC grade and stage, and significantly correlated with poor disease-free survival (DFS). Overexpression of SNHG3 can inhibit miR-214-3p expression, resulting in upregulation of its downstream target gene ASF1B. ASF1B was positively correlated with immune infiltration. Decreased ASF1B can significantly inhibit the expression of CD86, CD8, STAT1, STAT4, CD68 and PD-1 in HCC cells. LncRNA SNHG3/miR-214-3p/ASF1B axis can promote HCC recurrence by regulating immune infiltration [102]. LncRNA AC099850.3 was highly expressed in HCC tissues, predicting poor prognosis of HCC patients. Down-regulation of AC099850.3 could significantly inhibit the proliferation and metastasis, and promote the apoptosis of HCC cells. PRR11 was identified as a target gene for AC099850.3, and AC099850.3 exerted an oncogenic role on the PRR11/PI3K/AKT axis. AC099850.3 was negatively correlated with NK cells, M2 macrophages, and CD8^+^ T cells in the TME, which may be responsible for its tumorigenicity. It is noteworthy that AC099850.3 was significantly positively correlated with key immune checkpoint molecules (PD-1, PD-L1, PD-L2 and CTLA-4), making AC099850.3 a potential target for HCC immunotherapy [69]. The expression of lncRNA PED1B-AS1 and hsa-miR-194-5p were up-regulated in HCC. PED1B-AS1 was positively correlated with PD-1 ligands (PD-Ls) and negatively correlated with miR-194-5p. PED1B-AS1 promoted the expression of PD-Ls by sponging miR-194-5p. HCC cells released exosomes containing PED1B-AS1 and the exosomal PCED1B-AS1 enhanced the expression of PD-Ls in recipient HCC cells, while inhibiting recipient T cells and macrophages. Finally, they observed PCED1B-AS1 promoted proliferation, colony formation in vitro and tumorigenesis in vivo [103].

Pseudogene RP11-424C20.2 were frequently upregulated in HCC and acted as a ceRNA to increase UHRF1 expression through sponging miR-378a-3p [104]. LncRNA KCNQ1OT1 and PD-L1 were highly expressed in sorafenib-resistant HCC tissues while miR-506 was opposite. Knockdown of KCNQ1OT1 sensitized sorafenib-resistant HCC cells to sorafenib, changed the TME and T-cell apoptosis. In addition, dual-luciferase reporter assay verified KCNQ1OT1 functioned as a competing ceRNA of miR-506, leading to upregulation of PD-L1 [105].

A recent study demonstrated that a novel polymeric nanoparticle was designed to simultaneously target the TIGIT/poliovirus receptor (PVR) and lncRNA ANRIL. The nanoparticle administration exhibited an increase in the percentages of NK cells and T cells and an inhibition of HCC in vivo by simultaneously inhibited the expression of miR-203a and its downstream genes. Thus, simultaneously targeting of TIGIT/PVR and lncRNA ANRIL provides a novel strategy for HCC [106]. LncRNA nuclear-enriched autosomal transcript 1 (NEAT1) and TIM-3 was highly expressed in peripheral blood mononuclear cells (PBMCs) of patients with HCC. Down-regulation of NEAT1 inhibited apoptosis of CD8^+^ T cell and enhanced the cytolysis activity of CD8^+^ T cell against HCC cells through the miR-155/TIM-3 pathway. The NEAT1/miR-155/TIM-3 axis may be an effective target to improve the efficacy of immunotherapy in HCC [79].

### Breast cancer

Breast cancer is a complex disease with distinct molecular subtypes primarily according to the status of estrogen receptor (ER), progesterone receptor (PR) and ERBB2 receptor (HER2) [107]. It is reported that lncRNA T-cell leukemia/lymphoma 6 (TCL6) is a tumor suppressor in human cancer. In breast cancer, low expression of TCL6 was associated with ER and PR status, and an independent factor of poor prognosis. In PR(−) patients, low TCL6 expression is associated with poor prognosis. While in Luminal B patients, TCL6 can predict worse survival. Further analysis found TCL6 interacts with immune infiltrating cells such as B cells, neutrophils, DCs, CD8^+^ T cells, and CD4^+^ T cells. TCL6 was also positively correlated with immune checkpoint molecules such as PD-1, PD-L1, PD-L2 and CTLA-4 [70]. Studies have shown that PD-L1 is overexpressed in different molecular subtypes of breast cancer patients and MDA-MB-231 cells. LncRNA XIST and TSIX are pivotal elements in X chromosome inactivation (XCI) as well as breast cancer. They were differentially expressed in different molecular subtypes of breast cancer, and the expression levels of XIST and TSIX were correlated with the expression level of PD-L1. These results had shed light on the role of lncRNAs XIST and TSIX as potential non-invasive immune markers in breast cancer [108].

LncRNA, long intergenic non-coding RNA for kinase activation (LINK-A) is involved in drug resistance and hypoxia in breast cancer. The expression of LINK-A was highly expressed in triple-negative breast cancer (TNBC) compared with non-TNBC samples and predicted poor survival of patients with breast cancer. In addition, high LINK-A expression in human breast cancer tissues exhibited low CD8^+^CD3^+^ lymphocyte infiltration, indicating LINK-A was correlated with an immunosuppressive microenvironment. LINK-A promoted crosstalk between phosphatidylinositol -(3,4,5) -triphosphate and inhibitory G-protein-coupled receptor (GPCR) pathways and attenuated protein kinase A (PKA)-mediated phosphorylation of E3 ubiquitin ligase TRIM71. Thus, LINK-A expression enhanced k48-polyubiquitin mediated degradation of antigen-peptide-loaded complex (PLC) and inherent tumor suppressors Rb and p53. Administration of LINK-A-locked nucleic acid or GPCR antagonist stabilizes PLC components, Rb and p53, and sensitize breast cancer cells to immune checkpoint inhibitors. It was worth noting that TNBC patients with elevated LINK-A and downregulated PLC components were resistant to PD-1 inhibitors. Therefore, the results provides a basis for LINK-A/PKA/TRIM71 signaling axis molecules serving as biomarkers for immunotherapy sensitivity in breast cancer [109]. GATA3-AS1 was demonstrated as an oncogenic lncRNA in TNBC and predicted poor prognosis of TNBC patients. Knockdown of GATA3-AS1 inhibited proliferation and migration of TNBC cells. GATA3-AS1 stabilized PD-L1 protein through the deubiquitination of PD-L1, which was mechanically facilitated through miR-676-3p/COPS5 axis. In addition, GATA3-AS1 destabilized GATA3 protein by promoting GATA3 ubiquitination [61].

Activation-induced cell death (AICD) is an important apoptotic process involved in the control of T cell-mediated immune responses in the maintenance of immunological tolerance [110]. NKILA is a lncRNA that interacts with NF-κB and modulates T cell sensitivity to AICD by inhibiting NF-κB activity in lung caner and breast cancer microenvironment. Overexpression of NKILA in tumor-specific CTL and TH1 cells was associated with their apoptosis and shorter patient survival in breast cancer. These findings highlight the importance of lncRNAs in determining tumor-mediated T cell AICDs and suggest that engineered lncRNAs may provide a novel antitumor immunotherapeutic target in adoptively metastatic T cells [111]. FENDRR is a recently discovered tumor suppressor lncRNA whose expression is associated with epigenetic regulation of target genes involved in tumor immunity. Breast cancer cells with high FENDRR expression levels that typically exhibited upregulation of immune-activating genes and MHC-I molecules. Mechanistically, regulation of FENDRR expression enhanced inflammatory and WNT signaling pathways in tumors. The data suggested that FENDRR improved cancer immunotherapy through regulation of the development of immune-related phenotypes in breast cancer [112]. Ting Ye et al. found the expression level of MIAT in breast cancer tissues was significantly higher than that in normal tissues or adjacent tissues. MIAT expression was significantly correlated with 13 types of TIICs (B cells, DCs, neutrophils, CD8^+^ T cells, and so on). What’s more, higher expression of MIAT exhibited better immunotherapy effect. MIAT may be a valuable non-invasive diagnostic biomarker and predictor of responsiveness to immunotherapy for breast cancer [113].

### Stomach and colorectal cancer (CRC)

LncRNAs are identified to be dysregulated in stomach adenocarcinoma and play an important role in tumor immunity balance. Super-enhancers (SEs) comprise large clusters of enhancers that highly enhance gene expression including lncRNA expression. The expression of SE-associated lncRNA TM4F1-AS1 was negatively correlated with the proportion of CD8^+^ T cells in stomach adenocarcinoma. TM4F1-AS1 inhibited T cell-mediated immunity and predicted immune response to immune checkpoint inhibitors. Experimental data confirmed that TM4SF1-AS1 was regulated by its super enhancer and involved in immune pathways and cancer-related pathways [114]. LncRNA SNHG15 was highly expressed in gastric cancer tissues. Overexpression of SNHG15 upregulated the expression of PD-L1 and contributed to immune escape of gastric cancer cells. The possible mechanism was that SNHG15 sponged miR-141 and relieved the suppression of miR-141 on PD-L1 [115].

Immune dysregulation exerts a key role in colorectal tumorigenesis and progression. TMB is a novel biomarker widely used to predict the responsiveness of cancer patients to immunotherapy. Increasing evidence indicate that patients with mismatch repair deficient (dMMR) or MSI-H can benefit from anti-PD-1 immunotherapy [116]. LncRNA expression pattern is correlated with TMB, which can be used as a classifier for predicting TMB in colon cancer patients [117]. Chengsheng Ding et al. systematically studied the expression pattern of lncRNAs and found that lncRNA MIR22HG had a tumor suppressive effect in CRC. The expression of MIR22HG in CRC was significantly decreased, which was mainly driven by copy number deletion. Reduced MIR22HG expression was significantly associated with poorer OS. Importantly, they found that MIR22HG expression was significantly associated with CD8A, and overexpression of MIR22HG enhanced the immunotherapy by triggering T cell infiltration [118]. Further, Rui Chen et al. constructed the lncRNAs and miRNAs network and found an immune-related lncRNA, MIR17HG was highly expressed in CRC. MIR17HG increased NF-κB/RELA expression by competitively sponging miR-375. In addition, MIR17HG was transcriptionally activated by RELA. Thereby, MIR17HG promoted CRC progression and metastasis though building a positive feedback with RELA. Furthermore, MIR17HG could directly bind to PD-L1 and upregulate PD-L1 expression, suggesting its oncogenic role in immunotherapy. Taken together, these findings suggest that MIR17HG may be a promising therapeutic target in CRC [38].

Cholesterol metabolism disorders are vital for the progression of CRC. Simvastatin is a well-established inhibitor of 3-hydroxy-3-methylglutaryl-coA reductase (HMGCR), a rate-limiting enzyme in the cholesterol biosynthesis. Jianming Li et al. elucidated the role of simvastatin in regulating immune checkpoints or lncRNAs mediated immune regulation of CRC. Simvastatin inhibited the expression of lncRNA SNHG29, which interacted with and led to phosphorylation and ubiquitination-mediated protein degradation of Yes-associated protein (YAP), causing the inhibition of PD-L1 expression and the enhancement of cytotoxic T lymphocyte (CTL) infiltration. Thus, simvastatin/SNHG29/PD-L1 axis is considered to be a potential immunotherapy target in CRC [119].

A competitive endogenous RNA (ceRNA) network focused on the potential mechanisms of lncRNAs-derived CD8^+^ T cell infiltration was constructed and verified in CRC cells. Among them, the expression of LINC00657 was higher in CRC cells than other tumor cells. They demonstrated that LINC00657 impaired the cytotoxicity of CD8^+^ T cells and was negatively correlated with CD8^+^ T cell infiltration. The ceRNA network was constructed by miRNA-1224-3p, miRNA-338-5p, SCD, ETS2, UBE2H and YY1. Furthermore, in the ceRNA network, immunosuppressive tumor marker CD155 was positively correlated with LINC00657. Thus, LINC00657 may play a pivotal role in CRC immune escape [120]. Another study also reported that LINC00657 exerted its oncogenic role in CRC. Expression of LINC00657 had a positive correlation with clinical stage, lymph node metastasis, and poor prognosis of CRC patients. Mechanically, LINC00657 sponged miR-203a, leading to upregulation of its downstream target gene and promotion of CRC stem cell-like cell invasion ability [121].

### Other tumors

FOXP4-AS1 expression is involved in multiple signaling pathways and has been reported in various human cancers. FOXP4-AS1 expression was significantly correlated with FIGO stage of ovarian serous cystadenocarcinoma (OVs) and was an independent prognostic factor for OS. High expression of FOXP4-AS1 was associated with activation of the PD-1 signaling pathway, CTLA-4 signaling pathway, B cell receptor signaling pathway, and apoptosis. FOXP4-AS1 expression was negatively correlated with immune cell markers such as DCs, cytotoxic cells and neutrophils [122].

The majority of glioblastoma (GBM) patients are resistant to anti-PD-1/PD-L1 therapy, and only a minority of patients respond to this immunotherapy [123]. Previous studies demonstrated that high level of PD-1 facilitated immune evasion in glioma patients. Polymerase 1 and transcript release factor (PTRF/Cavin-1) was found to correlate with immunosuppression in GBM. Studies have shown that PTRF stabilized lncRNA NEAT1, induced the expression of PD-L1 and the activity of NF-κB signal pathway, and promoted immune escape in GBM. By inhibiting UBXN1 expression via NEAT1, PTRF enhanced PD-L1 transcription through promoting NF-κB activity. Finally, PTRF promoted immune escape of GBM cells though regulating PD-1 binding and PD-L1 mediated T cell toxicity [124].

LncRNA prostate cancer-associated transcript 6 (PCAT6) has been reported as an oncogene in many cancers. Jianfei Tu et al. observed elevated expression of PCAT6 in macrophages in patients with cholangiocarcinoma (CCA). Knockdown of PCAT6 significantly triggered an immune response and reduced tumor growth in vivo. In addition, overexpression of PCAT6 led to M2 polarization of THP-1-differentiated macrophages. MiR-326 was predicted and demonstrated to be a target of PCAT6. Meanwhile, the increase of PCAT6 has been demonstrated to induce the accumulation of ROS, mitochondria and metabolic dysfunction in macrophages. RohA acted as a downstream target of miR-326. The results highlighted the important role of PCAT6/miR-326/RohA axis in the immune response of macrophages and suggested that PCAT6 is a potential biomarker for immunotherapy of CCA [125].

## Conclusion and future perspectives

Recently, lncRNAs have been extensively reported in immunotherapy, which makes us realize the importance of lncRNAs in regulating immune escape of tumor cells. However, it is only the tip of the iceberg. This article reviewed the mechanisms of the regulation of lncRNAs and mechanisms of the action of lncRNAs, important immune checkpoints related signaling pathways, as well as the role of lncRNAs in regulating immune checkpoints, TME, tumor immune escape, and immune monitoring in different cancers.

**Fig. 5:**
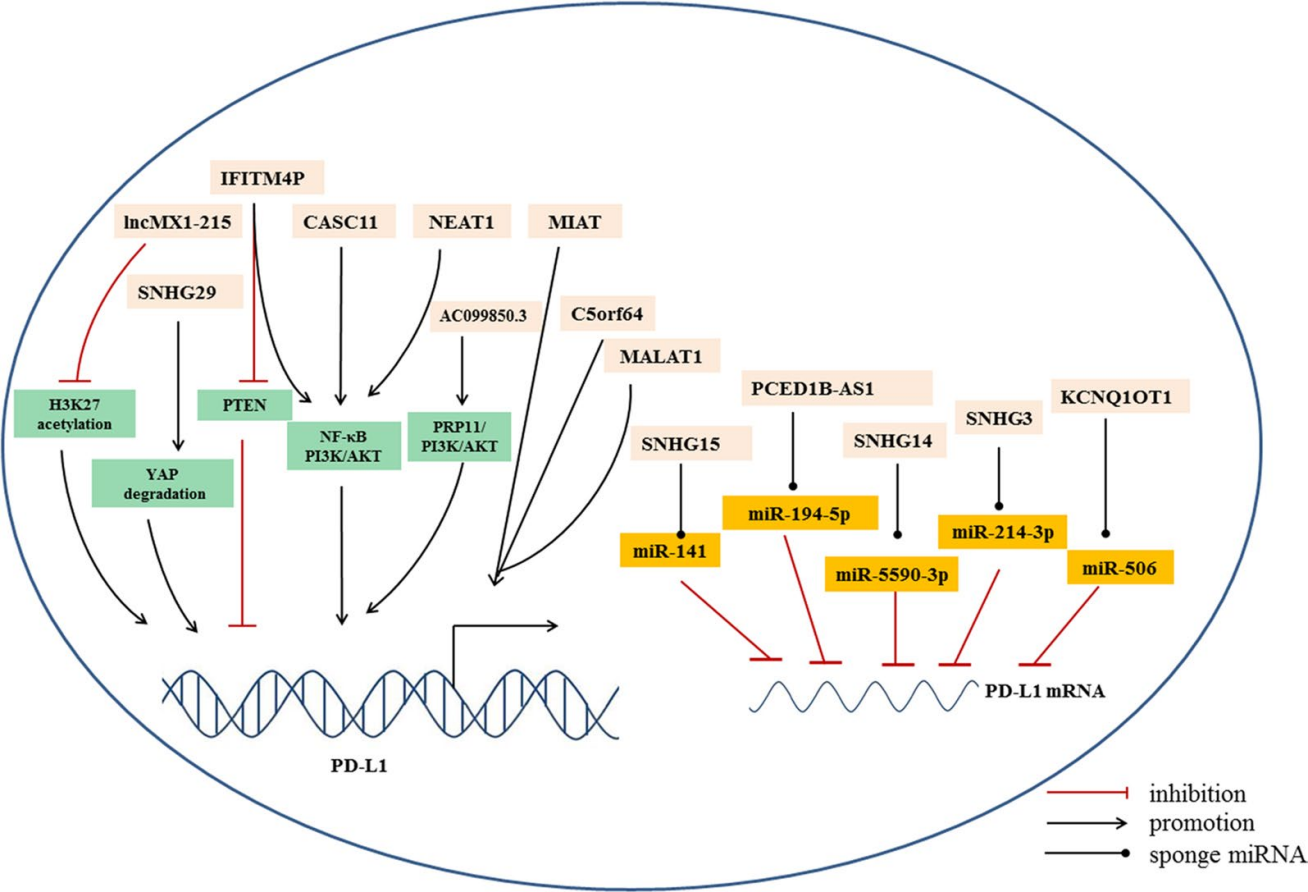
Multiple lncRNAs regulate the expression of PD-L1

In the literatures included in this review, we find that most immune-related lncRNAs contribute to immune escape through the following ways (Table 1). Firstly, lots of lncRNAs have been demonstrated to regulate the expression of key immune checkpoints, PD-1/PD-L1, CTLA-4, TIGIT, and TIM-3. Especially, many of them were validated to regulate the expression of PD-L1 transcriptionally or post-transcriptionally (Fig. 5). However, the detailed molecular mechanism of lncRNA-involved modulation of immune checkpoint pathways need to be further elucidated. One possible post-transcriptional regulatory mechanism is the lncRNA-miRNA network in PD-1/PD-L1 pathway. LncRNAs can sponge miRNAs as ceRNA to upregulate downstream target gene PD-L1, which contributing to immune evasion. Secondly, several lncRNAs exert their roles in immunotherapy through modulating T cells. Under normal physiological conditions, T cells recognize cancer cells, infiltrate at the tumor site, and kill tumor cells by cytotoxic effect. However, in pathological conditions and metastatic tumors, T cells in the TME exhibit an exhausted state, leading to primary resistance to immunotherapy. It was found that lncRNAs inhibit the antitumor activity, decreased proportion and induced exhaustion of CD8^+^ T cells. Finally, lncRNAs play an important role in modulating oncogenic signaling pathways involved in tumor proliferation and invasion, such as NF-κB, PI3K/AKT, Wnt/β-catenin pathways.

These studies provide a basis for us to explore lncRNAs as new biomarkers and therapeutic targets for immunotherapy. It is still necessary to further study and explore how lncRNA affects the tumor microenvironment and regulates the function of tumor immune cells. Although several clinical trials are being carried out on whether serum lncRNAs can be used as potential biomarkers in lung cancer diagnosis (NCT03830619), the diagnostic value and clinic utility of CCAT1 in colorectal cancer (NCT04269746), and so on (clinicalTrials.gov). Clinical trials of lncRNA as a regulator of PD-1/PD-L1 pathway in tumor immunotherapy have not been carried out. It is far from taking lncRNAs into the clinical application. One of the greatest challenges is developing a delivery system to deliver lncRNAs efficiently and with lasting effects to specific organs [9]. A more pronounced understanding of functions of lncRNAs and mechanisms of lncRNAs in immunology is urgent needed, which may be a promising and interesting research direction in the future.

## Data Availability

Not applicable.

## Abbreviations

lncRNAs: Long non-coding RNAs
CART: Chimeric antigen receptor T-cell immunotherapy
CTLA-4: Cytotoxic T-lymphocyte-associated protein 4
PD-1: The programmed cell death 1
PD-L1: Programmed cell death ligand 1
LAG-3: Lymphocyte-activation gene-3
TIGIT: T cell immunoreceptor with immunoglobulin and immunoreceptor tyrosine-based inhibitory motif domain
TIM3: T cell immunoglobulin- and mucin-domain-containing molecule 3
HOTAIR: HOX transcript antisense RNA
Xist: X-inactive specific transcript
NBAT-1: Neuroblastoma associated transcript-1
MALAT1: Metastasis-associated lung adenocarcinoma transcript 1
GR: Glucocorticoid receptor
DBD: DNA-binding domain
GREs: GR binds to glucocorticoid response elements
hESCs: Human embryonic stem cells
ITIM: Immune receptor tyrosine-based inhibitory motifs
ITSM: Immune receptor tyrosine-based switch motifs
NK cells: Natural killer cells
DCs: Dendritic cells
SHP-2: Src homologous region 2 domain
IFN-γ: Interferon-γ
NFAT: Nuclear factor of activated T cells
FoxO1: FoxO transcription factors
IRF9: IFN regulator 9
APC: Antigen presenting cells
mTOR: Mammalian target of rapamycin
Tregs: Regulatory T cells
TILs: Tumor-infiltrating lymphocytes
PVRs: Poliovirus receptors
HNSCC: Head and neck squamous cell carcinoma
MDSCs: Bone marrow-derived suppressor cells
NPC: Nasopharyngeal carcinoma
EMT: Epithelial–mesenchymal transition
ZEB1: Zinc finger E-box binding homeobox 1
SASH1: SAM and SH3 domain containing1
OSCC: Oral squamous cell carcinoma
NSCLC: Non small cell lung cancer
TMB: Tumor mutation burden
LUAD: Lung adenocarcinoma
STAT3: Signal transduction and transcription activator 3
HCC: Hepatocellular carcinoma
DFS: Disease-free survival
ER: Estrogen receptor
PR: Progesterone receptor
TNBC: Triple-negative breast cancer
CRC: Colorectal cancer
GBM: Glioblastoma
CCA: Cholangiocarcinoma
